# Supplementary material for: Laboratory mice engrafted with natural gut microbiota possess a wildling-like phenotype
Source: Nat Commun. 2025 Jun 12;16:5301. doi: 10.1038/s41467-025-60554-2 (PMC12162856; doi:10.1038/s41467-025-60554-2)
Supplement: Supplementary file 1 — Supplementary Information [file 41467_2025_60554_MOESM1_ESM.pdf]

Supplementary Fig. 1

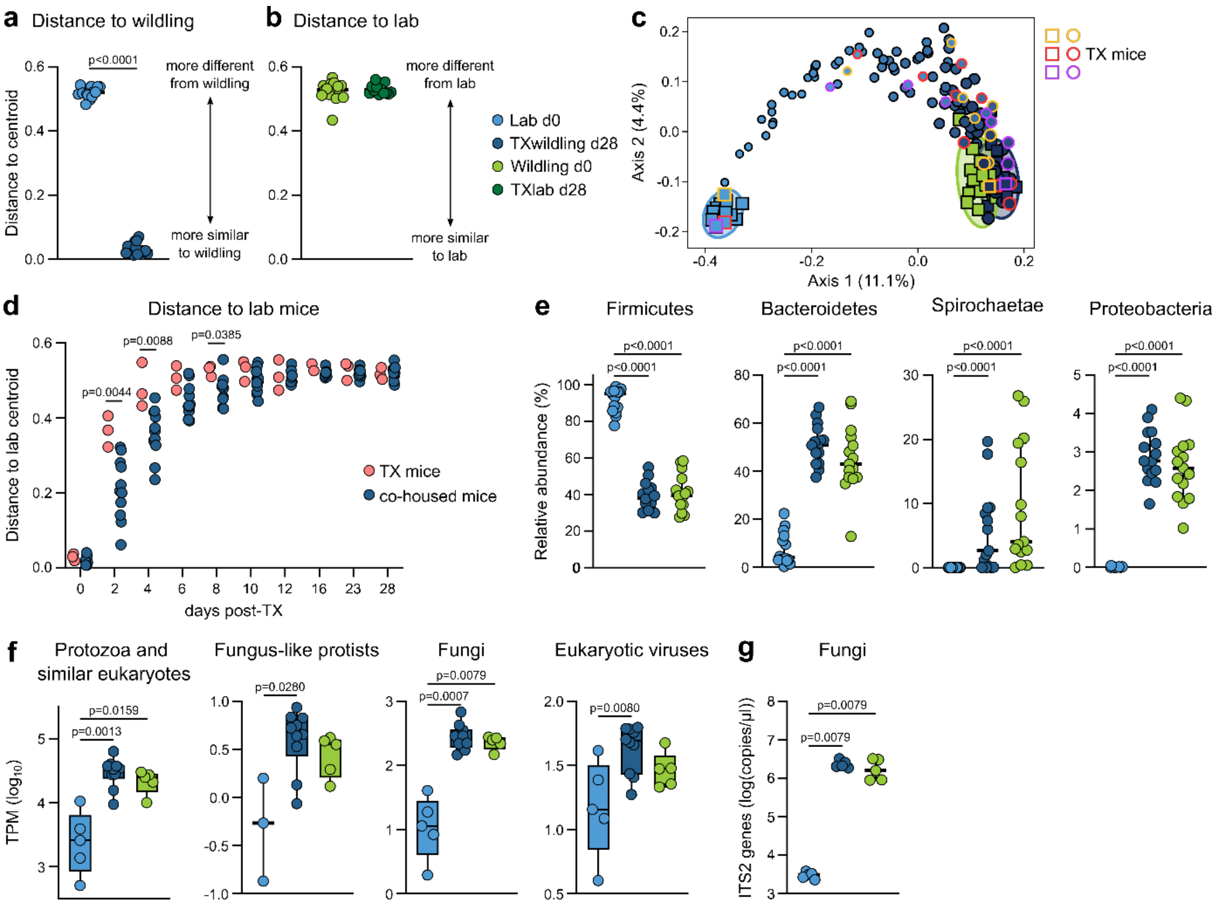

Figure S1: Natural gut microbiota of wildlings outcompete lab microbiota. (a) Graph shows the distance of lab mice (day 0 post-TX) or TXwildlings (day 28 post-TX) to the centroid of the wildling donors in the PCoA shown in Figure 1b. (b) Graph shows the distance of wildlings (day 0 post-TX and TXlab mice (day 28 post-TX) to the centroid of the lab donor mice in the PCoA shown in Figure 1d. (c) The graph shown in Figure 1b with those mice highlighted that received the microbiota transplantation. (d) The distance of each mouse at each timepoint to the centroid of the lab mice is calculated. The graph compares mice that received transplantation to those that were co-housed. (e) The relative abundance of the top four most abundant phyla in lab mice, TXwildlings and wildling is shown. (f) Shotgun sequencing data was analyzed for the presence of non-bacterial microorganisms. (g) Quantification of fungi by qPCR for ITS gene copies. Data are from three independent experiments (a-e) or one experiment (f-g) with n=5 (a-e, g) or n=5-10 (f) per group. Median ± IQR is shown, box plots show the median, interquartile range, and full data spread via whiskers. Statistical significances were tested by unpaired, two-tailed Mann-Whitney U tests, p<0.05 is shown. Source data are provided as a Source Data file.

Supplementary Fig. 2

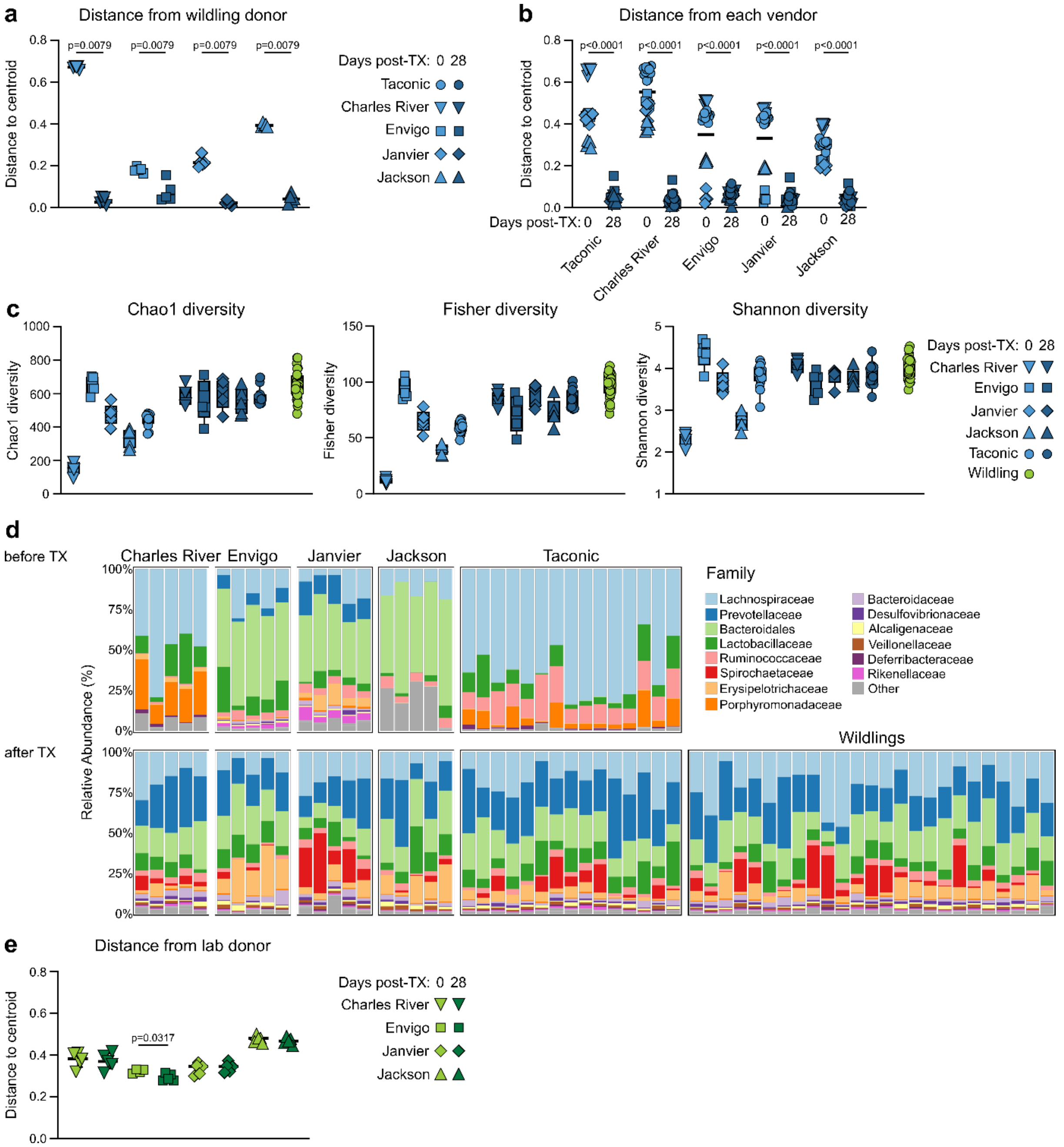

Figure S2: Natural gut microbiota of wildlings outcompete lab microbiota from different vendors. (a) Graph shows the distance of lab mice of different donors before and after transplantation to the centroid of the wildling donors. (b) The distance of each lab mouse before or after transplantation to the centroid of the indicated vendor at the respective day is shown. (c) Alpha diversity measures (Chao1, Fisher, Shannon) were calculated for lab microbiota of the different vendors before and 28 days post-transplantation with wildling fecal microbiota and compared to the alpha diversity of wildlings. (d) Relative abundances at the family level were calculated of the lab microbiota of the different vendors before and after transplantation as well as for wildlings. (e) Graph shows the distance of wildling mice before and after transplantation with microbiota of different lab vendors to the centroid of the respective donors. Data are from one (Charles River, Envigo, Janvier, Jackson), three (Taconic) or four (wildlings) independent experiments n=5 per group. Median is shown, box plots show the median, interquartile range, and full data spread via whiskers. Statistical significances were tested by unpaired, two-tailed Mann-Whitney U tests, $p<0.05$ is shown. Source data are provided as a Source Data file.

Supplementary Fig. 3

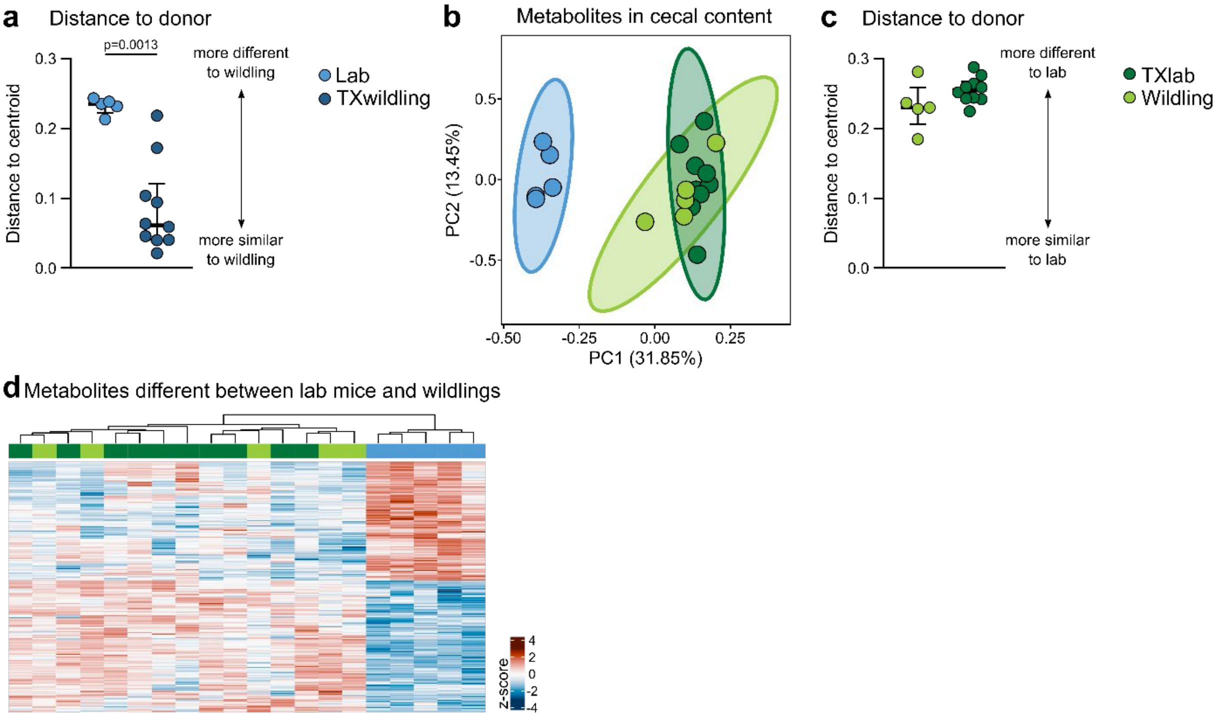

Figure S3: The global gut metabolomics profile of TXlab mice remains stable. A global metabolomics analysis was performed on the cecal content of wildlings, TXlab mice, and lab mice. (a) The distance of wildlings and TXlab mice to the centroid of the lab mice is depicted. (b) PCA of all detected metabolites was used to compare lab mice with TXlab mice and wildlings. (c) The distance of lab mice and TXwildlings to the centroid of wildlings in the PCA in Figure 2a is shown. (d) The heatmap shows the metabolites that were significantly different between lab mice and wildlings. Color code depicts the row-wise scaled (z-score) metabolite intensity. Median ± IQR is shown. Statistical significances in (a) and (c) were tested by unpaired, two-tailed Mann-Whitney U tests, p<0.05 is shown. Source data are provided as a Source Data file.

Supplementary Fig. 4

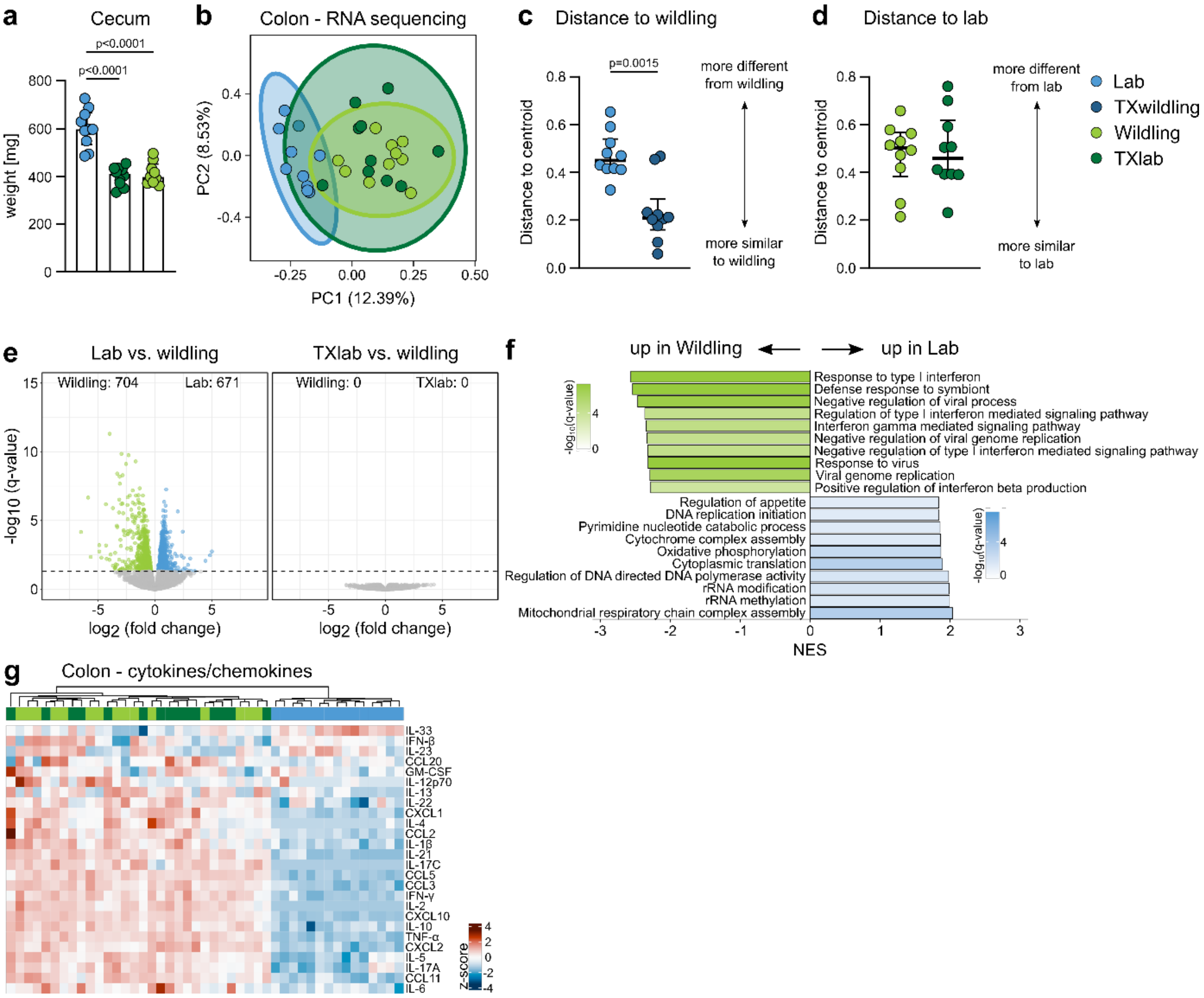

Figure S4: TXLab mice remain wildlings. Wildlings were engrafted with fecal material from lab mice, resulting in the TXlab mouse group, which was compared to lab mice and wildlings. (a) The cecum weight was measured and compared among the three groups. (b-f) The colon was harvested from mice of the three groups and subjected to RNA sequencing. Data was analyzed by PCA with ellipses showing a 95% confidence interval (b), the gene expression was compared between lab mice and wildlings or TXlab mice and wildlings (e), or GSEA was performed between lab mice and wildlings using Gene Ontology (Biological Processes aspect) (f). (c-d) Graphs show the distance of lab mice and TXwildlings (c) or wildlings and TXlab mice (d) to the centroid of the respective donors in the PCAs in Figure 3b and Figure S3b. (Eg) Cytokines and chemokines were analyzed in colon tissues of mice from the three groups by a multiplex MSD assay. Color code depicts the row-wise scaled (z-score) cytokine concentration. All cytokines that were above the detection limit in >50% of the samples are shown in the heatmaps. Data are from two (b-f) or three (a, g) independent experiments with n=5 per group. Median ± IQR is shown. Statistical significances were tested by unpaired, two-tailed Mann-Whitney U tests, $p<0.05$ is shown. Source data are provided as a Source Data file.

Supplementary Fig. 5

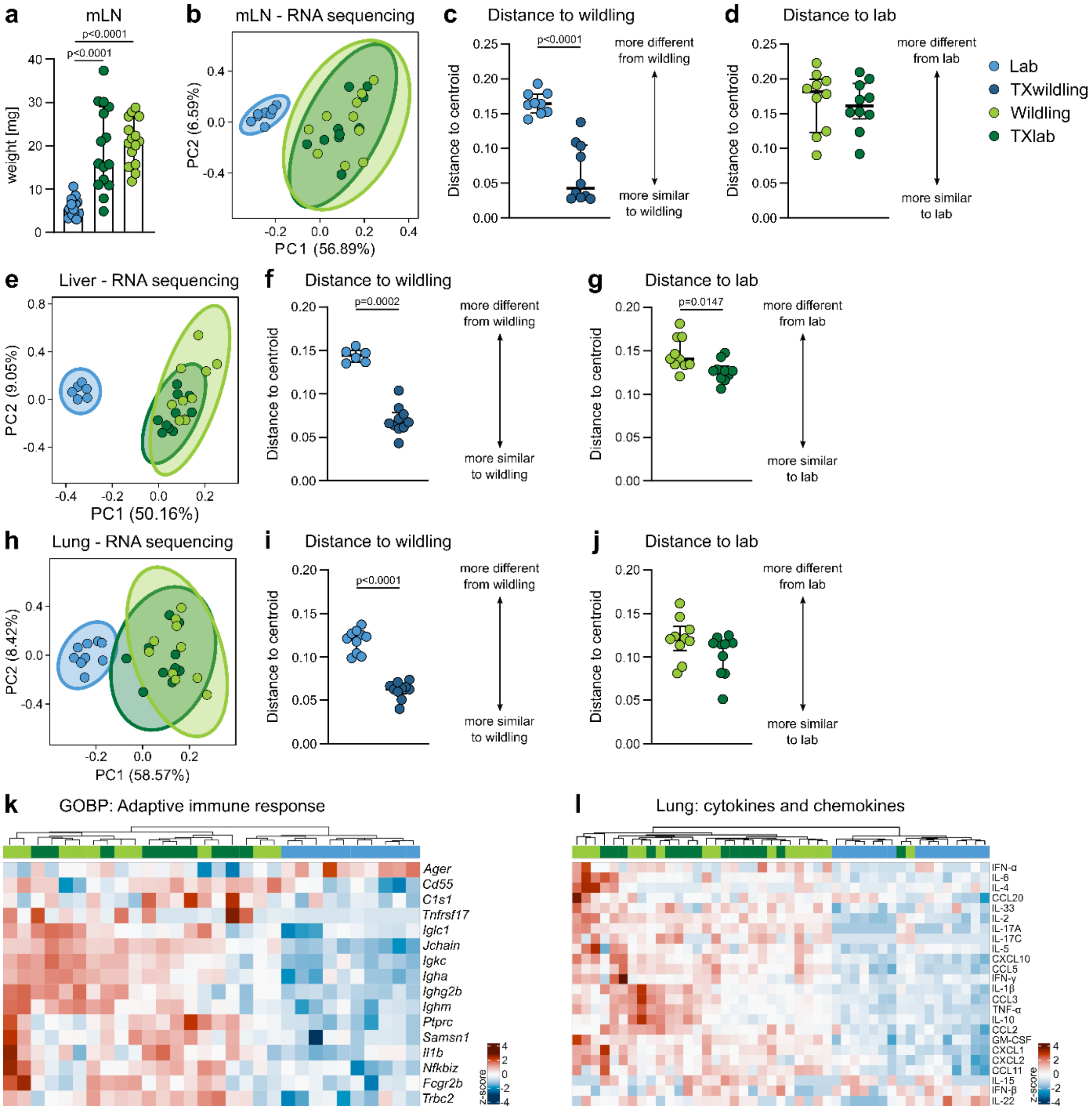

Figure S5: Analysis of mLN, liver, and lung tissue in lab mice, TX lab mice and wildlings. (a) The size of mLN of lab mice, TXlab mice and wildlings is shown. (b-d) They were subjected to RNA sequencing and PCA analysis was performed including the genes that were significantly different between lab mice and wildlings. (c) The distance between lab mice or TXwildlings to the centroid of the wildlings is shown (corresponding to Figure 4b) (d) The distance between wildlings and TXlab mice to the centroid of the lab mice is shown. (e-g) Livers of lab mice, TX lab mice and wildlings were subjected to RNA sequencing. (e) PCA analysis shows the genes significantly differently regulated between lab mice and wildlings. (f) The distance between lab mice or TXwildlings to the centroid of the wildlings is shown (corresponding to Figure 4c) (g) The distance between wildlings and TXlab mice to the centroid of the lab mice is shown. (h-j) The transcriptome of the lungs of lab mice, TXlab mice and wildlings was analyzed by RNA sequencing. (h) PCA using the genes significantly regulated between lab mice and wildlings compares the three groups. (i) The distance between lab mice or TXwildlings to the centroid of the wildlings is shown (corresponding to Figure 4g) (j) The distance between wildlings and TXlab mice to the centroid of the lab mice is shown. (k) The heatmap shows the significantly regulated genes ($q<0.05$) of the ”adaptive immune response” gene sets. Color code depicts the row-wise scaled (z-score) RNA normalized expression. (l) Cytokines and chemokine concentration was analyzed in lung tissue by a multiplex MSD assay. Color code depicts the row-wise scaled (z-score) cytokine concentration. All cytokines, that were above the detection limit in >50% of all samples are shown in the heatmap. Ellipses in (b), (e), and (h) represent the 95% confidence interval. Data are from two (b-l) or three (a) independent experiments with n=5 per group. Median ± IQR is shown. Statistical significances were tested by unpaired, two-tailed Mann-Whitney U tests. Source data are provided as a Source Data file.

Supplementary Fig. 6

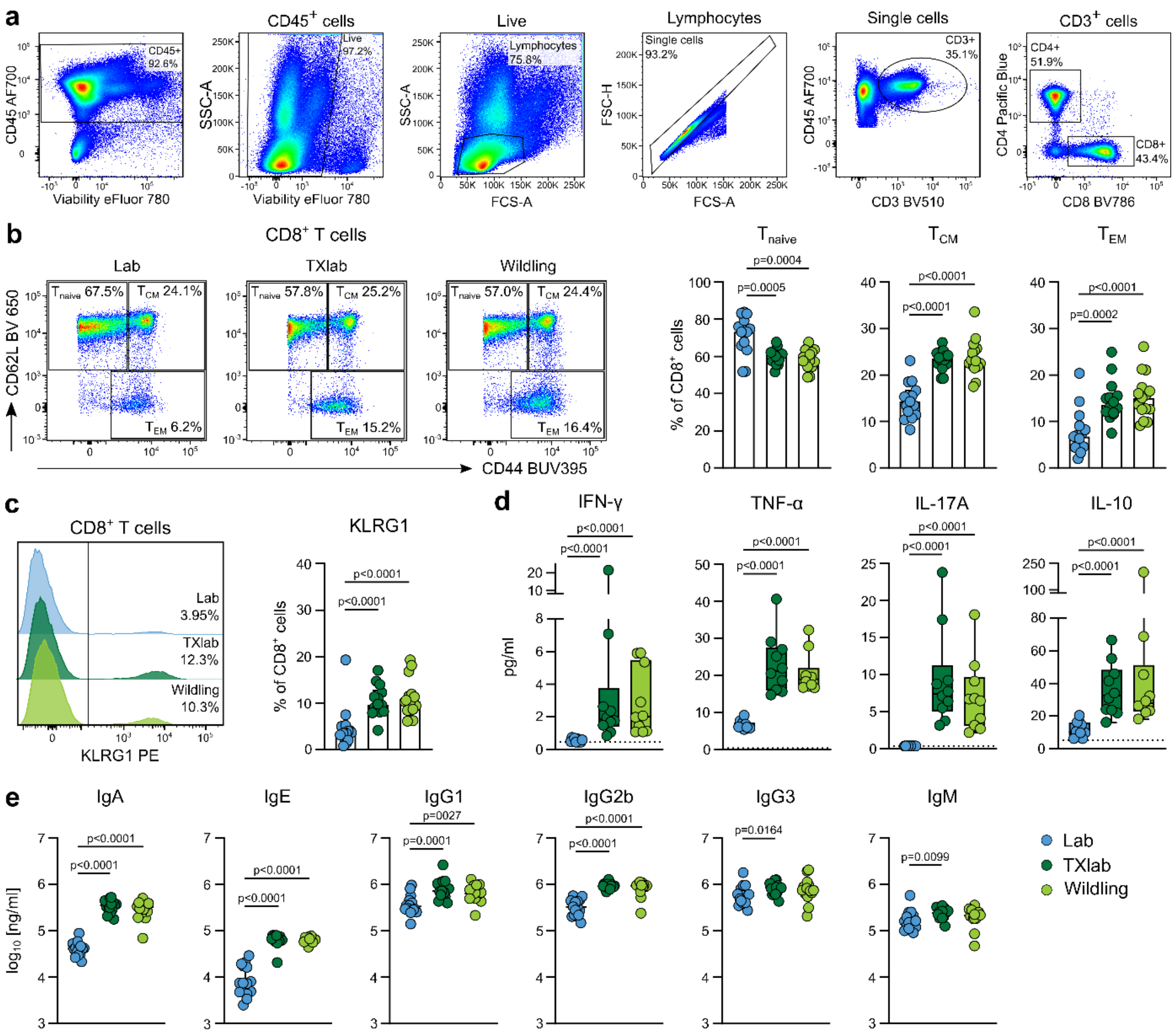

Figure S6: Systemic immunity of TXlab mice is not altered. (a-c) Blood cells were isolated, analyzed by flow cytometry, and gated for live CD45⁺CD3⁺CD8⁺ cells as shown in the gating strategy in (a). (b) T_{naive}, T_{CM}, and T_{EM} were identified by their expression of CD44 and CD62L as shown in the left panel and analyzed as the percentage of total CD8⁺ cells. (c) KLRG1⁺ cells were identified (left panel) and analyzed as percentage of total CD8⁺ cells. (d) Serum cytokine concentrations were quantified using a multiplex MSD assay. (e) Antibody subclasses were measured in the serum of lab mice, TXlab mice, and wildlings. The shown data are from two (d) or three (b-c, e) independent experiments with n=5 mice per group. Dashed lines indicate the detection limit. Median ± IQR is shown, box plots show the median, interquartile range, and full data spread via whiskers. Statistical significance was tested by unpaired, two-tailed Mann-Whitney U test, p<0.05 is shown. Source data are provided as a Source Data file.

Supplementary Table 1

Group non-bacterial microorganisms	Taxonomic Classification form Contig Annotation Tool (CAT)
Fungi	Eukaryota;Ascomycota;Saccharomycetes;Saccharomycetales;Saccharomycetaceae;Arxiozyma;Arxiozyma heterogenica
Fungi	Eukaryota;Chytridiomycota;Neocallimastigomycetes;Neocallimastigales;Neocallimastigaceae;Neocallimastix;no support
Fungi	Eukaryota;Ascomycota;Saccharomycetes;Saccharomycetales;Saccharomycetaceae;no support;no support
Fungi	Eukaryota;Mucoromycota;Glomeromycetes;Glomerales;Glomeraceae;Rhizophagus;Rhizophagus irregularis
Fungi	Eukaryota;Basidiomycota;Agaricomycetes;Boletales;Paxillaceae;Paxillus;no support
Fungi	Eukaryota;Chytridiomycota;Neocallimastigomycetes;Neocallimastigales;Neocallimastigaceae;Neocallimastix;Neocallimastix lanati (nom. inval.)
Fungi	Eukaryota;Ascomycota;Saccharomycetes;no support;no support;no support;no support
Fungi	Eukaryota;Mucoromycota;Glomeromycetes;no support;no support;no support;no support
Fungi	Eukaryota;Chytridiomycota;Neocallimastigomycetes;Neocallimastigales;Neocallimastigaceae;Piromyces;Piromyces finnis
Fungi	Eukaryota;Chytridiomycota;Neocallimastigomycetes;Neocallimastigales;Neocallimastigaceae;no support;no support
Fungi	Eukaryota;Microsporidia;NA;NA;Spragueidae;Spraguea*;Spraguea lophii*
Fungi	Eukaryota;Chytridiomycota;Neocallimastigomycetes;Neocallimastigales;Neocallimastigaceae;Anaeromyces;Anaeromyces robustus
Fungi	Eukaryota;Chytridiomycota;Chytridiomycetes;NA;NA;Blyttiomyces;Blyttiomyces helicus
Fungi	Eukaryota;Zoopagomycota;Harpellomycetes;Harpellales;Legeriomycetaceae;Smittium;Smittium mucronatum
Fungi	Eukaryota;Chytridiomycota;Neocallimastigomycetes;Neocallimastigales;Neocallimastigaceae;Piromyces;Piromyces sp. E2
Fungi	Eukaryota;Basidiomycota;Tremellomycetes;Filobasidiales;Filobasidiaceae;Naganishia;Naganishia vaughanmartiniae
Fungi	Eukaryota;Ascomycota;no support;no support;no support;no support;no support
Fungi	Eukaryota;Microsporidia;NA;NA;Pleistophoridae;Vavraia;Vavraia culicis*
Fungi	Eukaryota;Microsporidia;NA;NA;NA;Dictyocoela;Dictyocoela muelleri
Fungi	Eukaryota;Chytridiomycota;Chytridiomycetes;Rhizophydiales;Terramycetaceae;Boothiomyces;Boothiomyces macroporosus
Protozoa and similar eukaryotes	Eukaryota;Parabasalia;NA;Tritrichomonadida;Tritrichomonadidae;Tritrichomonas;Tritrichomonas foetus
Protozoa and similar eukaryotes	Eukaryota;Apicomplexa;Aconoidasida;Haemosporida;Plasmodiidae;Plasmodium;Plasmodium yoelii
Protozoa and similar eukaryotes	Eukaryota;Parabasalia;no support;no support;no support;no support;no support
Protozoa and similar eukaryotes	Eukaryota;Parabasalia;NA;Tritrichomonadida;no support;no support;no support
Protozoa and similar eukaryotes	Eukaryota;Parabasalia;NA;Trichomonadida;Trichomonadidae;Trichomonas;Trichomonas vaginalis
Protozoa and similar eukaryotes	Eukaryota;Parabasalia;NA;Tritrichomonadida;Dientamoebidae;Histomonas;Histomonas meleagridis*
Protozoa and similar eukaryotes	Eukaryota;Evosea;NA;Mastigamoebida;Entamoebidae;Entamoeba;Entamoeba invadens
Protozoa and similar eukaryotes	Eukaryota;Parabasalia;NA;Tritrichomonadida;Tritrichomonadidae;Tritrichomonas*;no support
Protozoa and similar eukaryotes	Eukaryota;Fornicata;NA;Diplomonadida;Hexamitidae;Spironucleus;Spironucleus salmonicida
Protozoa and similar eukaryotes	Eukaryota;Fornicata;NA;Diplomonadida;Hexamitidae;Giardia;Giardia intestinalis
Protozoa and similar eukaryotes	Eukaryota;Fornicata;NA;Diplomonadida;Hexamitidae;Giardia*;no support
Protozoa and similar eukaryotes	Eukaryota;Preaxostyla;NA;Oxymonadida;Polymastigidae;Monocercomonoides;Monocercomonoides exilis
Protozoa and similar eukaryotes	Eukaryota;Preaxostyla;NA;Oxymonadida;Streblomastigidae;Streblomastix*;Streblomastix strix*

Protozoa and similar eukaryotes	Eukaryota;Ciliophora;Oligohymenophorea;Hymenostomatida;Tetrahymenidae;Tetrahymena;Tetrahymena thermophila
Protozoa and similar eukaryotes	Eukaryota;Fornicata;NA;Diplomonadida;Hexamitidae*;no support;no support
Protozoa and similar eukaryotes	Eukaryota;Apicomplexa;Aconoidasida;Haemosporida;Plasmodiidae;Plasmodium;Plasmodium berghei
Protozoa and similar eukaryotes	Eukaryota;Apicomplexa;Aconoidasida;Haemosporida;Plasmodiidae;Plasmodium;no support
Protozoa and similar eukaryotes	Eukaryota;Ciliophora;Heterotrichea;Heterotrichida;Stentoridae;Stentor;Stentor coeruleus
Protozoa and similar eukaryotes	Eukaryota;Fornicata;NA;Diplomonadida;Hexamitidae;Giardia;Giardia muris
Protozoa and similar eukaryotes	Eukaryota;Fornicata;NA;NA;NA;Kipferlia;Kipferlia bialata*
Eukaryotic viruses	Viruses;Cressdnaviricota;Arfiviricetes;Cirlivirales;Circoviridae;NA;Rodent circovirus
Eukaryotic viruses	Viruses;Cressdnaviricota;no support;no support;no support;no support;no support
Eukaryotic viruses	Viruses;Hofneiviricota;Faserviricetes;Tubulavirales;Inoviridae;NA;Inoviridae sp.
Eukaryotic viruses	Viruses;Artverviricota;Revtraviricetes;Ortervirales;Retroviridae;Intracisternal A-particles;Mouse Intracisternal A-particle
Fungus-like protists	Eukaryota;Oomycota;NA;Saprolegniales;Saprolegniaceae;Aphanomyces;Aphanomyces euteiches

Supplementary Table 1: Non-bacterial constituent of the microbiota.
The table shows the taxonomic information of the top 20 non-bacterial microorganism per group that were found in wildlings and TXwildlings.

Supplementary Table 2

	Lab mice (n=10-120)*	TXwildlings (n=20)**	Wildlings (n=20)**
**Bacteria**			
Bordetella bronchiseptica	No	No	No
Citrobacter rodentium	No	No	No
Clostridium piliforme	No	No	No
Corynebacterium kutscheri	No	No	No
Filobacterium rodentium (CAR Bacillus)	No	No	No
Helicobacter species	No	**Yes**	**Yes**
Helicobacter bilis	No	**Yes**	**Yes**
Helicobacter ganmani	No	**Yes**	**Yes**
Helicobacter hepaticus	No	No	No
Helicobacter mastomyrinus	No	**Yes**	**Yes**
Helicobacter rodentium	No	No	No
Helicobacter typhlonius	No	**Yes**	**Yes**
Klebsiella oxytoca	No	No	No
Klebsiella pneumoniae	No	No	No
Mycoplasma pulmonis	No	No	No
Proteus mirabilis	No	No	No
Pseudomonas aeruginosa	No	No	No
Rodentibacter pneumotropicus	No	No	No
Rodentibacter heylii	No	No	No
Salmonella species	No	No	No
Staphylococcus aureus	No	No	No
Streptobacillus moniliformis	No	No	No
Streptococcus pneumoniae	No	No	No
Streptococcus sp. β-hemolytic Group A	No	No	No
Streptococcus sp. β-hemolytic Group B	No	No	No
Streptococcus sp. β-hemolytic Group C	No	No	No
Streptococcus sp. β-hemolytic Group G	No	No	No
**Viruses**			
Ectromelia virus	No	No	No
Mouse Rotavirus (EDIM)	No	No	No
Hantaan	No	No	No
Lymphocytic Choriomeningitis Virus (LCMV)	No	No	No
Mouse Adenovirus (MAdV-1)	No	No	No
Mouse Adenovirus (MAdV-2)	No	**Yes**	**Yes**
Murine Cytomegalovirus (MCMV)	No	No	No
Mouse Hepatitis Virus (MHV)	No	No	No
Murine Norovirus (MNV)	No	**Yes**	**Yes**
Mouse Parvoviruses (MPV)	No	No	No
Minute virus of mice (MVM)	No	No	No
Pneumonia Virus of Mice (PVM)	No	No	No
Reovirus (REO3)	No	No	No
Sendai Virus	No	No	No

Theiler's murine encephalomyelitis virus (TMEV)	No	**Yes**	**Yes**
**Protozoa**			
Chilomastix species	No	**Yes**	**Yes**
Cryptosporidium species	No	No	No
Eimeria species	No	No	No
Entamoeba muris	No	**Yes**	**Yes**
Giardia muris	No	No	No
Spironucleus muris	No	**Yes**	**Yes**
Tritrichomonas muris	No	**Yes**	**Yes**
Pinworm species			
Aspiculuris tetraptera	No	**Yes**	**Yes**
Syphacia muris	No	No	No
Syphacia obvelata	No	No	No

Supplementary Table 2: Pathogen profile of lab mice, TXwildlings and wildlings.
* The hygiene standard of lab mice was assessed and reported by the mouse vendor (Taconic Biosciences).
** The hygiene standard of TXwildlings and wildlings was determined by utilizing PCR (the Mouse 3R Quarantine Annual SOPF, MCMV, and Hantaan) on pooled samples and Global Serology on individual samples. A microorganism was considered present, if it was identified through at least one of the two independent methodologies.

Supplementary Table 3

Fluorophor	Antigen	Klon	Dilution	Manufacturer	Catalogue number
BUV395	CD44	IM7	1:100	BD	740215
Pacific Blue	CD4	GK1.5	1:1000	BioLegend	100428
BV510	CD3	145-2C11	1:50	BioLegend	100353
BV650	CD62L	MEL-14	1:800	BioLegend	104453
BV786	CD8	53-6.7	1:1500	BD	563332
PE	KLRG1	2F1/KLRG1	1:800	BioLegend	138407
AF700	CD45.2	104	1:400	BioLegend	109822

Table S3: Detailed antibody list.
