# Supplementary material for: Laboratory mice engrafted with natural gut microbiota possess a wildling-like phenotype
Source: Nat Commun. 2025 Jun 12;16:5301. doi: 10.1038/s41467-025-60554-2 (PMC12162856; doi:10.1038/s41467-025-60554-2)
Supplement: Supplementary file 2 — Description of Additional Supplementary Files [file 41467_2025_60554_MOESM2_ESM.pdf]

Description of Additional Supplementary Files

File Name: Supplementary Data 1.
Description: Metabolomics data of all samples shown in Figure 2 and Supplementary Figure 3.

File Name: Supplementary Data 2:
Description: Cytokine data of the heatmaps shown in Figure 3e and Supplementary Figure 4g (colon) and Figure 4j and Supplementary Figure 5l (lung).
