# Supplementary material for: Laboratory mice engrafted with natural gut microbiota possess a wildling-like phenotype
Source: Nat Commun. 2025 Jun 12;16:5301. doi: 10.1038/s41467-025-60554-2 (PMC12162856; doi:10.1038/s41467-025-60554-2)
Supplement: Supplementary file 5 — Reporting Summary [file 41467_2025_60554_MOESM5_ESM.pdf]

nature portfolio

Corresponding author(s): Prof. Dr. Stephan P. Rosshart

Last updated by author(s): Apr 4, 2025

Reporting Summary

Nature Portfolio wishes to improve the reproducibility of the work that we publish. This form provides structure for consistency and transparency in reporting. For further information on Nature Portfolio policies, see our Editorial Policies and the Editorial Policy Checklist.

Statistics

For all statistical analyses, confirm that the following items are present in the figure legend, table legend, main text, or Methods section.

n/a	Confirmed	
☐	☒	The exact sample size (*n*) for each experimental group/condition, given as a discrete number and unit of measurement
☐	☒	A statement on whether measurements were taken from distinct samples or whether the same sample was measured repeatedly
☐	☒	The statistical test(s) used AND whether they are one- or two-sided<br>*Only common tests should be described solely by name; describe more complex techniques in the Methods section.*
☒	☐	A description of all covariates tested
☒	☐	A description of any assumptions or corrections, such as tests of normality and adjustment for multiple comparisons
☐	☒	A full description of the statistical parameters including central tendency (e.g. means) or other basic estimates (e.g. regression coefficient) AND variation (e.g. standard deviation) or associated estimates of uncertainty (e.g. confidence intervals)
☐	☒	For null hypothesis testing, the test statistic (e.g. *F*, *t*, *r*) with confidence intervals, effect sizes, degrees of freedom and *P* value noted<br>*Give P values as exact values whenever suitable.*
☒	☐	For Bayesian analysis, information on the choice of priors and Markov chain Monte Carlo settings
☒	☐	For hierarchical and complex designs, identification of the appropriate level for tests and full reporting of outcomes
☒	☐	Estimates of effect sizes (e.g. Cohen's *d*, Pearson's *r*), indicating how they were calculated

Our web collection on statistics for biologists contains articles on many of the points above.

Software and code

Policy information about availability of computer code

Data collection	Flow cytometry data was collected on LSR Fortessa with FACSDiva software.<br>MSD Assays were analysed with the help of DISCROVERY WORKBENCH version 4.0<br>ELISA data was collected by SparkConotrol magellan software version 2.2.<br>Histology pictures were quality controlled using Phenochart version 1.2., exported using inForm version 2.8.0.
Data analysis	Flow cytometry data was analyzed using FlowJo software version 10.6.2 (BD).<br>Raw RNA sequencing reads were analysed using CLC Genomics Workbench 20.0.4 “Demultiplex QIAseq UPX<br>742 3' reads” tool and bcl2fastq2 software and UMI-tools. Alignment was done using STAR (version 2.7.11a). Post-alignment deduplication was done with UMI-tools (version 1.1.4) and followed by gene-level quantification with htseq (version 2.0.3).<br>Analysis and visualization of metabolomics, RNA sequencing and cytokines/chemokine measurements were performed using R (version 4.3.2) with the edgeR, clusterProfiler, MSigDB databases.<br>16S rRNA sequencing raw reads were demultiplexed by idemp and processed using the USEARCH pipeline version 11.9.667. Following Taxonomic assignment by Constax 2.0.20 with the Greengenes2 2022.10 database. WGS reads assembled by Megahit 1.13, contigs classified by CAT 6.01.<br>Histology pictures were analysed with QuPath version 0.3.2.

For manuscripts utilizing custom algorithms or software that are central to the research but not yet described in published literature, software must be made available to editors and reviewers. We strongly encourage code deposition in a community repository (e.g. GitHub). See the Nature Portfolio guidelines for submitting code & software for further information.

Data

Policy information about availability of data

All manuscripts must include a data availability statement. This statement should provide the following information, where applicable:

- Accession codes, unique identifiers, or web links for publicly available datasets
- A description of any restrictions on data availability
- For clinical datasets or third party data, please ensure that the statement adheres to our policy

• The RNA sequencing data generated in this study have been deposited in the Gene Expression Omnibus (GEO) database under accession code GSE287225 [https://www.ncbi.nlm.nih.gov/geo/query/acc.cgi?acc=GSE287225].
• The RNA sequencing data used for creating Figure 6d are available in the GEO database under accession code GSE27272 [https://www.ncbi.nlm.nih.gov/geo/query/acc.cgi?acc=GSE27272].
• The 16S rRNA and ITS2 gene amplicon and shotgun sequencing data generated in this study have been deposited in the BioProject database under accession code PRJEB84922 [https://www.ncbi.nlm.nih.gov/bioproject/?term=PRJEB84922].
• The metabolomics data generated in this study (Figure 2) are provided in the Supplementary Information.
• The cytokine data generated in this study (Figure 3e and Figure 4j) are provided in the Supplementary Information.
• Source Data are provided with this paper.

Research involving human participants, their data, or biological material

Policy information about studies with human participants or human data. See also policy information about sex, gender (identity/presentation), and sexual orientation and race, ethnicity and racism.

Reporting on sex and gender	*Use the terms sex (biological attribute) and gender (shaped by social and cultural circumstances) carefully in order to avoid confusing both terms. Indicate if findings apply to only one sex or gender; describe whether sex and gender were considered in study design; whether sex and/or gender was determined based on self-reporting or assigned and methods used.*<br>*Provide in the source data disaggregated sex and gender data, where this information has been collected, and if consent has been obtained for sharing of individual-level data; provide overall numbers in this Reporting Summary. Please state if this information has not been collected.*<br>*Report sex- and gender-based analyses where performed, justify reasons for lack of sex- and gender-based analysis.*
Reporting on race, ethnicity, or other socially relevant groupings	*Please specify the socially constructed or socially relevant categorization variable(s) used in your manuscript and explain why they were used. Please note that such variables should not be used as proxies for other socially constructed/relevant variables (for example, race or ethnicity should not be used as a proxy for socioeconomic status).*<br>*Provide clear definitions of the relevant terms used, how they were provided (by the participants/respondents, the researchers, or third parties), and the method(s) used to classify people into the different categories (e.g. self-report, census or administrative data, social media data, etc.)*<br>*Please provide details about how you controlled for confounding variables in your analyses.*
Population characteristics	*Describe the covariate-relevant population characteristics of the human research participants (e.g. age, genotypic information, past and current diagnosis and treatment categories). If you filled out the behavioural & social sciences study design questions and have nothing to add here, write "See above."*
Recruitment	*Describe how participants were recruited. Outline any potential self-selection bias or other biases that may be present and how these are likely to impact results.*
Ethics oversight	*Identify the organization(s) that approved the study protocol.*

Note that full information on the approval of the study protocol must also be provided in the manuscript.

Field-specific reporting

Please select the one below that is the best fit for your research. If you are not sure, read the appropriate sections before making your selection.

☒ Life sciences ☐ Behavioural & social sciences ☐ Ecological, evolutionary & environmental sciences

For a reference copy of the document with all sections, see nature.com/documents/nr-reporting-summary-flat.pdf

Life sciences study design

All studies must disclose on these points even when the disclosure is negative.

Sample size	Sample size for mouse studies was chosen according to institutional directives and in accordance with the 3Rs (Replacement, Reduction and Refinement) guiding principles underpinning the humane use of animals in research, but no statistical analyses were performed to predetermine the sample sizes. The sample size of 10 or 15 animals in most groups results from 5 mice being in one experiment. So 10 or 15 mice results from two or three independent experiments, respectively.

Data exclusions	Data that failed quality controls were excluded.
Replication	All attempts at replication were successful, with multiple mice in each group. Some animal experiments were not replicated, but all experimental groups consisted of at least 5 animals with similar results. Also, we observed similar results with mice from different vendor, indicating well-replicable results.
Randomization	Mice were randomly allocated to different treatments.
Blinding	Blinding was not applied due to hygiene standards.

Reporting for specific materials, systems and methods

We require information from authors about some types of materials, experimental systems and methods used in many studies. Here, indicate whether each material, system or method listed is relevant to your study. If you are not sure if a list item applies to your research, read the appropriate section before selecting a response.

Materials & experimental systems

n/a	Involved in the study	
☐	☒	Antibodies
☒	☐	Eukaryotic cell lines
☒	☐	Palaeontology and archaeology
☐	☒	Animals and other organisms
☒	☐	Clinical data
☒	☐	Dual use research of concern
☒	☐	Plants

Methods

n/a	Involved in the study	
☒	☐	ChIP-seq
☐	☒	Flow cytometry
☒	☐	MRI-based neuroimaging

Antibodies

Antibodies used	CD44 BUV395 clone: IM7 supplier: BD catalog number: 740215 Dilution:1:100 Lot: 2003548<br>CD4 Pacific Blue clone: GK1.5 supplier: BioLegend catalog number: 100428 Dilution: 1:1000 Lot: B347337<br>CD3 BV510 clone: 145-2C11 supplier: BioLegend catalog number: 100353 Dilution: 1:50 Lot: B340821<br>CD62L BV650 clone: MEL-14 supplier: BioLegend catalog number: 104453 Dilution: 1:800 Lot: B337524<br>CD8 BV786 clone: 53-6.7 supplier: BD catalog number: 563332 Dilution: 1:1500 Lot: 1221630<br>KLRG1 PE clone: 2F1/KLRG1 supplier: BioLegend catalog number: 138407 Dilution: 1:800 Lot: B357617<br>CD45.2 AF700 clone: 104 supplier: BioLegend catalog number: 109822 Dilution: 1:400 Lot: B336461
Validation	All antibodies were obtained by commercial vendors and we based specificity on descriptions and information provided in the available data sheets and articles provided by the manufacturers.<br>Antibody titration was performed on mouse splenocytes with regards to discrimination of positive and negative cell populations.

Animals and other research organisms

Policy information about studies involving animals; ARRIVE guidelines recommended for reporting animal research, and Sex and Gender in Research

Laboratory animals	Laboratory mice used in this study were C57BL/6NTac (MPF) ordered from Taconic, Denmark if not indicated otherwise. For one experiment, laboratory mice with SPF microbiota were purchased from The Jackson Laboratory (C57BL/6J), Charles River (C57BL/6NCrl), Envigo (C57BL/6JRccHsd), and Janvier Labs (C57BL/6JRj). Wildling C57BL/6NTac mice were created by embryo transfer of laboratory mouse into pseudopregnant wild mice (Rosshart et al, Science (2019)) and bred locally. All mice used were 12-16 weeks old.
Wild animals	This study did not involve wild animals
Reporting on sex	Due to the 1:4 co-housing setting in our model, all experiments were performed with female mice only.
Field-collected samples	This study did not involve field-collected samples
Ethics oversight	Experiments were performed in accordance with the guidelines of the Federation for Laboratory Animal Science Associations and the national animal welfare body. They were in compliance with the German animal protection law and were approved by the animal welfare committee of the Regierungspräsidium Freiburg (permit G-21/030).

Note that full information on the approval of the study protocol must also be provided in the manuscript.

Plants

Seed stocks

Report on the source of all seed stocks or other plant material used. If applicable, state the seed stock centre and catalogue number. If plant specimens were collected from the field, describe the collection location, date and sampling procedures.

Novel plant genotypes

Describe the methods by which all novel plant genotypes were produced. This includes those generated by transgenic approaches, gene editing, chemical/radiation-based mutagenesis and hybridization. For transgenic lines, describe the transformation method, the number of independent lines analyzed and the generation upon which experiments were performed. For gene-edited lines, describe the editor used, the endogenous sequence targeted for editing, the targeting guide RNA sequence (if applicable) and how the editor was applied.

Authentication

Describe any authentication procedures for each seed stock used or novel genotype generated. Describe any experiments used to assess the effect of a mutation and, where applicable, how potential secondary effects (e.g. second site T-DNA insertions, mosiacism, off-target gene editing) were examined.

Flow Cytometry

Plots

Confirm that:

- [x] The axis labels state the marker and fluorochrome used (e.g. CD4-FITC).
- [x] The axis scales are clearly visible. Include numbers along axes only for bottom left plot of group (a 'group' is an analysis of identical markers).
- [x] All plots are contour plots with outliers or pseudocolor plots.
- [x] A numerical value for number of cells or percentage (with statistics) is provided.

Methodology

Sample preparation

Blood was collected by cardiac puncture into EDTA-coated monovettes (Sarstedt). For erythrocyte lysis, the blood was added to 10 ml of 1x RBC Lysis Buffer (BioLegend) and incubated for 4 min. After addition of 40 ml of PBS, the cell suspension was centrifuged (336 ×g, 12 min, 4°C) and the cells were counted.

Instrument

LSRFortessa (BD, Germany)

Software

FlowJo_v10.7.1

Cell population abundance

Analysis was performed on bulk cells isolated from the blood

Gating strategy

Gating on CD45+ cells, exclusion of dead cells, lymphocytes gated on FSC-A and SSC-A, doublet exclusion on FSC-A and FCS-H, gating on CD3+ cells, gating on CD8+ CD4- cells.

- [x] Tick this box to confirm that a figure exemplifying the gating strategy is provided in the Supplementary Information.
